# Fitness Costs Predict Emotional, Moral, and Attitudinal Inbreeding Aversion

**DOI:** 10.3389/fpsyg.2016.01860

**Published:** 2016-11-24

**Authors:** Florence Lespiau, Gwenaël Kaminski

**Affiliations:** ^1^UMR 5163 Cognition, Langues, Langage, Ergonomie - Laboratoire Travail et Cognition, Université de ToulouseToulouse, France; ^2^Institut Universitaire de FranceParis, France

**Keywords:** kinship, inbreeding, degree of involvement, degree of relatedness, coresidence

## Abstract

In terms of sexual intercourse, the very last people we think about are our kin. Imagining inbreeding intercourse, whether it involves our closest kin or not, induces aversion in most people who invoke inbreeding depression problems or cultural considerations. Research has focused on the disgust felt when facing inbreeding intercourse between close kin but little is known about other responses. In this study, we considered the influence of fitness costs on aversive reactions by including disgust and emotional reaction as well as moral judgment and attitudes toward inbreeding: higher costs should induce a stronger aversive reaction. The fitness costs were manipulated by two factors: (i) the degree of the participants' involvement in the story (themselves, a sib or an unknown individual), and (ii) the degree of relatedness between the two inbreeding people (brother/sister, uncle-aunt/niece-nephew, cousin). To test this hypothesis, 140 women read and assessed different inbreeding stories varying in the fitness costs incurred. Findings showed that the higher the fitness costs were, the greater the aversive reaction was in an overall way. First, our results fitted with previous studies that tested the influence of fitness costs on disgust. Second, and more interestingly, findings went further by examining overall aversion, showing that fitness costs could influence emotions felt as well as attitudes and behaviors toward inbreeding people. The higher the fitness costs were, the less inbreeding people were perceived as moral and the more they were considered as a nuisance. However, results regarding avoidance were more nuanced.

## Introduction

Just try to imagine:

“*Alan and Brenda are brother and sister. They are both of legal age (about the same age) and celibate. They went to Egypt for their vacation. One night, they decided to have sexual intercourse for fun. Brenda had been taking a contraceptive pill regularly for a long time. In addition, they used a condom in order to avoid any risk. They both liked making love together but decided not do it again. After that night, although they felt closer to each other, they decided to keep it secret.”*

What kind of emotion do you feel? Probably disgust or a more general aversive reaction. Then, try to explain why you feel these emotions. Do not just think “I am disgusted because Alan and Brenda are an inbreeding couple, and that is the way I am supposed to feel because most people are averse to incest.” Try to give some different arguments. “They will have a baby with birth defects.” No, they used two contraceptive methods. “Incest is prohibited.” Actually, having inbreeding intercourse is not prohibited in most countries (unlike incestuous marriage). “Inevitably, they will regret their intercourse.” No, they feel closer to each other. “One of them forced the other.” No, they are both of legal age and it was something they did for fun. “They will give a bad example to society.” No, they chose to keep it secret. You are probably thinking about arguments that justify your first emotion. Most of the time, biases in reasoning imply that we do not seek the truth but we look for arguments to substantiate our initial intuition (or emotion; Haidt, 2001). Moral judgments are not only the result of reasoning but also a combination of heuristics such as experiencing disgust or empathy (both of which are more or less unconscious). Thus, inbreeding intercourse is thought to be morally wrong, although people can argue about it. Therefore, the most important predictor of moral judgment and behaviors is emotional reaction (Haidt, 2007).

It is a fact that most people experience disgust and aversion faced with inbreeding people. Evidence supporting this has been shown in different research using fictitious stories about third-party inbreeding behaviors. Indeed, the disgust felt when facing third-party inbreeding intercourse is shaped by the disgust felt when we are personally involved in inbreeding (Lieberman et al., 2003, 2007). Thanks to egocentric empathy, each of us reacts to third-party inbreeding as if it was his/her own behavior to a lesser extent (Fessler and Navarrete, 2004). The strength of the disgust felt varies greatly from one individual to another (Park et al., 2008). Studies have highlighted different individual factors that can modulate aversion. First, as minimum parental investment is much larger for a woman than for a man (Trivers, 1972), it was supposed that women are more sensitive to disgust when it comes to inbreeding intercourse (Fessler and Navarrete, 2004; Park, 2008; Antfolk et al., 2012a,b; Marcinkowska et al., 2013). Second, during their high fertility period (when the risks of procreation are higher), women feel more disgusted faced with inbreeding people than during their low fertility period (DeBruine et al., 2005; Antfolk et al., 2014). Third, when someone lived with an other-sex sibling during childhood, s/he is supposed to feel greater aversion faced with inbreeding people (Lieberman et al., 2003, 2007; Fessler and Navarrete, 2004; the “Westermarck effect”). A study about facial attractiveness using a different paradigm showed similar results: women who have lived with a brother are less influenced by the self-resemblance bias for male attractiveness than women without a brother (DeBruine et al., 2011). These results allowed us to elaborate a gradient showing the variations in disgust that people feel faced with inbreeding individuals and to make hypotheses: on one hand, women in their high fertility period who lived with an other-sex child would feel more intense disgust, on the other hand, men who did not live with other children during childhood would be less disgusted.

Now, try to imagine the same story but with the following variant:

“*You have just been told that your sister and brother, who are both of legal age (about the same age) and celibate, went to Austria for the vacation. One night, they decided to have sexual intercourse for fun…”*

Do you feel the same emotion when reading this story compared to the first one? Probably not. Your disgust is probably stronger and you feel several negative emotions. You may say that this behavior is completely immoral and you may be thinking about some way to punish your siblings. This time, the story is actually different because it involves your own kin.

The psychological mechanisms involved in avoiding intercourse with our close kin must be sensitive to the costs incurred by inbreeding intercourse in which we are personally involved—since it implies a reduction in our direct fitness (inbreeding depression). And, these mechanisms must also take into account the costs incurred by inbreeding intercourse between two of our kin—since intercourse between our own kin implies a reduction in our indirect fitness (Hamilton, 1964; Haig, 1999). Thus, in the case of inbreeding intercourse involving a third-party, the intensity of indignation and the degree of aversion are linked to the costs incurred by the intercourse. The costs are even higher when the individuals involved are our close kin. Finally, the higher the degrees of involvement and relatedness regarding the sexual act are, the higher the costs are. The influence of the degrees of involvement and relatedness in inbreeding intercourse on disgust was recently examined (Antfolk et al., 2012b). This study used a third-party inbreeding paradigm as well as stories involving the participant or a kin. Effectively, the findings showed that unrelated third-party inbreeding stories elicited less disgust than related third-party descriptions and stories personally involving the participants.

Most research on aversive reactions to inbreeding intercourse focused on emotions felt, mostly disgust, and did not take into account the other aspects of aversive reactions (Haidt, 2003; Fessler and Navarrete, 2004). Effectively, because of the strong motivational component of disgust to incite individuals to avoid and exclude the disgusting object, the reluctant function of disgust is said to have been co-opted by natural selection, particularly regarding inbreeding aversion (Haidt, 2003; Lieberman et al., 2003, 2007). Little research has directly examined judgment about the morality of inbreeding behavior or explored its perceived nuisance, and avoidance and punishment of inbreeding people. One study, for example, investigated avoidance, the disgust felt and the punitive reaction faced with inbreeding people (Fessler and Navarrete, 2004). Therefore, in order to benefit from a more sensitive vision of aversive reaction, in addition to negative emotional reactions, we also examined moral judgment and attitudes of avoidance regarding inbreeding people. In this way, we hope to answer a question that is more difficult than it initially seems: how do people behave faced with inbreeding couples?

We conducted an experiment which allowed us to vary the fitness costs incurred by inbreeding intercourse. Based on their actual kin, participants could be presented with an inbreeding story involving themselves, a kin or a non-kin third-party. The degree of relatedness between inbreeding people also varied (intercourse could be between brother/sister, or uncle-aunt/niece-nephew, or cousins). Using items involving disgust, negative emotions, moral judgment, nuisance, and avoidance attitude as dependent measures, we tested three predictions. First, we assumed that because of higher fitness costs, inbreeding aversion should be greater for higher degrees of involvement in inbreeding intercourse whatever the dependent variables (greatest when the participant herself is involved, and greater when a participant's sib is involved compared to a third-party). Second, we expected greater aversion when the degree of relatedness between the two individuals involved was higher. Finally, according to the Westermarck effect (Lieberman et al., 2007; Lieberman and Smith, 2012), we predicted that aversion should be greater for individuals who had lived with other-sex siblings.

## Materials and methods

### Participants

The sample consisted of 159 female participants recruited from an email list of graduate students at the University of Toulouse. This anonymous study was conducted online using a Qualtrics survey and lasted about 30 min. All subjects gave written informed consent in accordance with the Declaration of Helsinki. Women who reported having personally experienced an incestuous relationship (*N* = 19) were excluded from the analysis because of possible bias (however, it is possible that other participants who experienced such intercourse but did not want to tell about it remain in our sample). Thus, the data obtained from 140 women (mean age: 24 years, ranging from 18 to 67 years; for socio-demographic details, see the Electronic Supplementary Material ESM 1) were used in our study. The participants were asked to report the number and sex of siblings, the duration of coresidence with siblings, the number of uncles/aunts and cousins, as well as the status of their relationship (single, in relationship, etc.).

### Procedure

The participants were presented with two stories which they had to judge. The first story involved inbreeding intercourse, the second non-inbreeding intercourse with exactly the same details (within-subject). The inbreeding story was presented before the non-inbreeding story to validate that the differences observed were due to the inbreeding feature. As we were not interested in the non-inbreeding story but in the inbreeding one, we did not want to risk creating a bias toward inbreeding stories. The non-inbreeding story was used to ensure that the general aversive reaction between stories was due to inbreeding and not to another feature of the story (e.g., intergenerational, sexual intercourse for fun, and just once). This was confirmed by our results: compared with non-inbreeding stories, inbreeding stories elicited stronger aversive reactions (*ps* < 0.001), i.e., higher negative emotions, avoidance and a feeling of nuisance as well as a lower sense of morality, and this was shown for all participants. We did not use the non-inbreeding stories in the analyses and, as they were presented second, they did not influence the participants' responses regarding inbreeding stories.

The fictional inbreeding story involved secret, safe, heterosexual and casual intercourse between two consenting individuals of at least 18 years old (as in the story described in the Section Introduction). We manipulated two factors: the degree of involvement and the degree of relatedness. The individuals described in the stories varied randomly in both their (1) degree of involvement [stories could involve the participant herself (high involvement), or her sib (intermediate involvement), or a third-party (low involvement)] and (2) the degree of relatedness (*r*) between the two people involved (brother/sister (*r* = 0.5), uncle-aunt/niece-nephew (*r* = 0.25), or cousins (*r* = 0.125) in intercourse). Based on the description of her actual relatives, each participant was presented with one of these stories, which allowed promoting greater fidelity of the participant's observed reactions (see ESM 2 in Supplementary Material for examples).

After reading this story, the participants had to report their reactions to it on a visual analog scale (from 0 to 100). Five variables were assessed. First, the participants had to indicate their level of (i) disgust felt (which was part of the negative emotions) to allow comparison between our data and those found in previous studies which generally used a Likert-type scale. Then, they had to rate (ii) the negative emotions felt, (iii) the perceived moral aspect, and (iv) the nuisance induced by the acts described for the characters' family and friends, for society and for the characters themselves. Finally, they answered items regarding (v) their avoidance attitude toward people involved in the inbreeding stories, such as changes in behavior concerning lending money and getting together with them (see ESM 3 in Supplementary Material for details and accessory variables).

### Statistical analyses

Generalized linear models (GLM) with a negative binomial distribution and a log link function were used to analyze observations with the Wald-test. We conducted an ordinal logistic regression with three categories on the disgust felt (see ESM 4 in Supplementary Material). Pre-planned linear contrast analyses were conducted to test the differences between each condition according to our assumptions. We also tested the influence of having lived with another child and the influence of the longest duration of coresidence with a same-sex sib or with an other-sex sib. Means and odds-ratios (OR) were computed, with 95% confidence intervals in brackets. Statistical analyses were conducted using SAS Version 9.2.

## Results

First, to replicate previous results, we tested the influence of the degrees of involvement, and relatedness on disgust alone. The degrees of involvement [$\chi_{\left(2\right)}^{2}$ = 10.73, *p* < 0.005] and relatedness [$\chi_{\left(2\right)}^{2}$ = 10.42, *p* = 0.005] strongly influenced the disgust felt. The higher the degrees of involvement and relatedness were, the greater the disgust was (OR = 4.28 [1.72–10.64]; $\chi_{\left(1\right)}^{2}$ = 9.76, *p* < 0.002 and OR = 3.82 [1.68–8.69]; $\chi_{\left(1\right)}^{2}$ = 10.24, *p* < 0.002, respectively). Moreover, findings showed an influence of having lived with another child [$\chi_{\left(2\right)}^{2}$ = 8.80, *p* = 0.012]. More precisely, the longer the duration of coresidence with a same-sex sibling was, the more the disgust felt was stronger (OR = 1.08 [1.02–1.14]; $\chi_{\left(1\right)}^{2}$ = 6.76, *p* = 0.009). However, the influence of the duration of coresidence with an other-sex sibling was not significant (OR = 1.03 [0.97–1.09]; $\chi_{\left(1\right)}^{2}$ = 0.93, *p* = 0.334).

**Figure 1:**
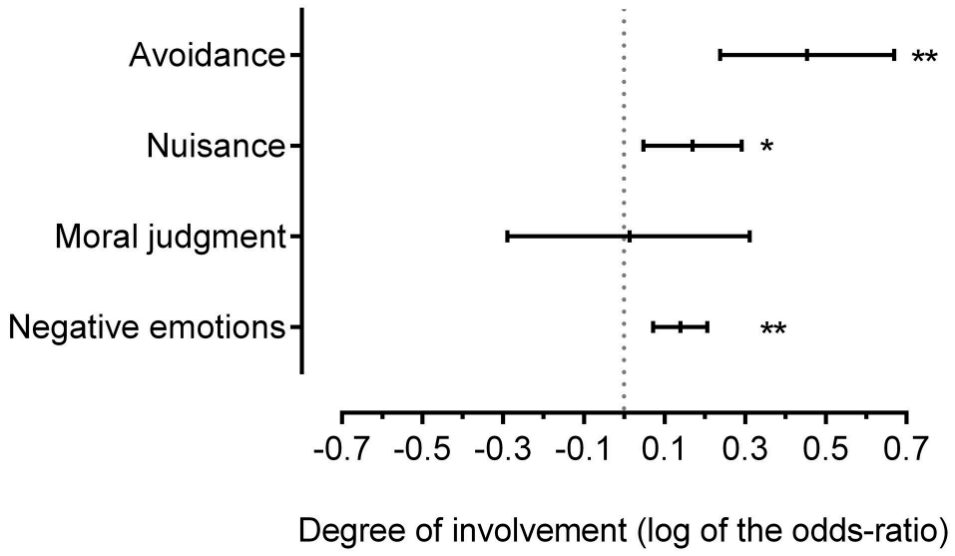
**Influence of the degree of involvement on aversive reactions (log of the odds-ratio, LOR and 95% confidence interval CI; the “avoidance” measure is a reversed one)**. The degree of involvement implies 3 ordinal modalities: participant herself (high degree of involvement), participant's sib (intermediate) and third-party (low). If LOR = 0: the degree of involvement does not affect the odds of outcome; LOR > 0: the degree of involvement is associated with higher odds of outcome; LOR < 0: the degree of involvement is associated with lower odds of outcome. ^*^p < 0.05; ^**^p < 0.005.

**Figure 2:**
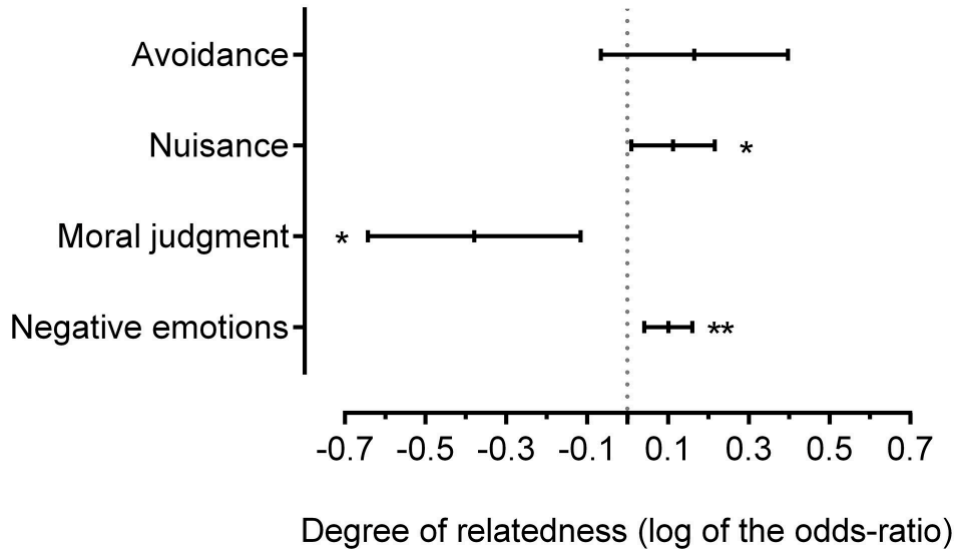
**Influence of the degree of relatedness on aversive reactions (log of the odds-ratio, LOR and 95% confidence interval CI; the “avoidance” measure is a reversed one)**. The degree of relatedness implies 3 ordinal modalities: brother/sister (*r* = 0.5), uncle-aunt/niece-nephew (*r* = 0.25) and cousins (*r* = 0.125) intercourse. If LOR = 0: the degree of relatedness does not affect the odds of outcome; LOR > 0: the degree of relatedness is associated with higher odds of outcome; LOR < 0: the degree of relatedness is associated with lower odds of outcome. ^*^p < 0.05; ^**^p < 0.005.

We then tested the influence of the degrees of involvement and relatedness on the general aversive reaction to inbreeding. According to the first prediction, the closer the participants are to the individuals involved, the greater the aversion would be. Effectively, the degree of involvement had a significant effect on each dependent variable except the moral aspect (see Table 1 for main effects and Figure 1 for contrasts). As expected, the higher the degree of involvement was, the higher the scores of negative emotions and nuisance were. Unexpectedly, when the degree of involvement was higher, participants avoided inbreeding people less. According to the second prediction, the closer the inbreeding people are, the greater the aversion would be. Effectively, the degree of relatedness had a significant effect on each dependent variable except avoidance (see Table 2 for main effects and Figure 2 for contrasts). As expected, the higher the degree of relatedness was, the higher the scores for negative emotions and nuisance were, and the lower the moral judgment score was.

**Table 1:** **Influence of the degree of involvement on aversive reactions**.

**Variables**	**High**		**Intermediate**		**Low**		**Wald statistics**	
	**M**	**CI**	**M**	**CI**	**M**	**CI**	**$\chi_{\left(2\right)}^{2}$**	***p***
Negative Emotions	42.64	[36.29–50.10]	39.99	[35.37–45.21]	30.95	[28.20–33.95]	18.42	0.0001
Moral Judgment	19.47	[11.06–34.27]	14.04	[8.47–23.26]	18.88	[12.99–27.42]	1.24	0.540
Nuisance	51.78	[38.15–70.28]	42.66	[32.78–55.52]	35.05	[27.56–44.56]	7.51	0.020
Avoidance	31.05	[19.31–49.93]	13.27	[8.08–21.79]	10.93	[7.37–16.19]	21.42	<0.0001

*Analysis are described with Mean (M) and 95% Confidence Interval (CI). Wald statistics test if at least one of the means is different from the two others*.

**Table 2:** **Influence of the degree of relatedness on aversive reactions**.

**Variables**	***r*** = **0.5**		***r*** = **0.25**		***r*** = **0.125**		**Wald statistics**	
	**M**	**CI**	**M**	**CI**	**M**	**CI**	**$\chi_{\left(2\right)}^{2}$**	***p***
Negative Emotions	41.74	[37.5–46.36]	38.22	[34.02–42.93]	33.08	[28.48–38.42]	10.69	0.005
Moral Judgment	12.08	[7.22–20.23]	14.74	[9.56–22.71]	28.97	[19.69–42.63]	10.14	0.006
Nuisance	47.38	[37.26–60.24]	44.72	[34.27–58.37]	36.53	[27.23–49.00]	4.80	0.090
Avoidance	16.30	[10.87–24.42]	24.83	[15.40–40.01]	11.13	[6.76–18.33]	9.09	0.010

*Analysis are described with Mean (M) and 95% Confidence Interval (CI). Wald statistics test if at least one of the means is different from the two others*.

Finally, regarding our third prediction, the aversion would be greater for individuals who had lived with an other-sex sib. Findings showed that the participants avoided less inbreeding people but tended to feel greater negative emotions when they had been living with a sibling (*N* = 124), regardless of their gender (Table 3). When we took into account the sib's gender (Table 4), the duration of coresidence with an other-sex sib only influenced moral judgment: the longer it was, the more the participants judged the behavior as immoral. Moreover, the longer the duration of coresidence with a same-sex sib was, the stronger the negative emotions were, and the harsher the moral judgment was.

**Table 3:** **Influence of having lived with (at least) one sib on aversive reactions**.

**Variables**	**Having lived**		**Not having lived**		**Wald statistics**	
	**M**	**CI**	**M**	**CI**	**$\chi_{\left(1\right)}^{2}$**	***p***
Negative Emotions	41.23	[38.8–43.81]	34.13	[28.27–41.19]	3.53	0.060
Moral Judgment	15.09	[11.26–20.22]	19.78	[11.75–33.30]	0.75	0.390
Nuisance	51.29	[46.57–56.48]	35.41	[22.63–55.41]	2.54	0.110
Avoidance	27.20	[22.13–33.43]	10.03	[5.09–19.74]	7.71	0.005

*Analysis are described with Mean (M) and 95% Confidence Interval (CI). Wald statistics test if one mean is different from the other*.

**Table 4:** **Influence of the longest coresidence duration with a same-sex sib and an other-sex sib on aversive reactions**.

**Variables**	**Same-sex sib**			**Wald statistics**		**Other-sex sib**			**Wald statistics**	
	**Estim**.	**OR**	**CI**	**$\chi_{\left(1\right)}^{2}$**	***p***	**Estim**.	**OR**	**CI**	**$\chi_{\left(1\right)}^{2}$**	***p***
Negative Emotions	0.01	1.01	[1.01–1.02]	4.16	0.041	0.01	1.01	[0.10–1.01]	1.22	0.269
Moral Judgment	−0.06	0.94	[0.90–0.99]	6.12	0.013	−0.04	0.96	[0.92–1.01]	3.77	0.052
Nuisance	0.01	1.01	[0.10–1.02]	2.15	0.143	0.01	1.01	[0.99–1.02]	0.40	0.523
Avoidance	−0.01	0.99	[0.96–1.02]	0.48	0.490	0.01	1.01	[0.98–1.04]	0.41	0.520

*Analysis are described with Estimate (Estim.), odds-ratios (OR), and 95% Confidence Interval (CI)*.

## Discussion

The present study further explored how people behave faced with inbreeding couples. With this aim, we varied the degree of the participants' involvement in the inbreeding story based on their actual relatives and the degree of relatedness between the two people involved. The results were clear: the higher the fitness costs were, the stronger general aversion was, not only the disgust felt but also the negative emotions felt in a more general way, the moral judgment, and the attitudes regarding nuisance and avoidance. These findings complement Antfolk et al. (2012b) and extend their results regarding the effects of fitness costs on the disgust felt to two other dimensions of aversive reactions to inbreeding people (attitudes and behaviors).

When imagining people in inbreeding intercourse, we feel negative emotions most of the time. We feel it more when these people are our own kin. Moreover, the ultimate aversion comes when we are personally involved in inbreeding. As expected, regarding the degree of involvement, the fitness costs influenced negative emotions, and the nuisance. Nevertheless, analysis showed an interesting unexpected fact: the more the participants were involved, the more they chose to continue their interactions with inbreeding people and did not exclude them. This finding could be explained by the fact that the higher the degree of involvement was, the closer the participants were, genetically speaking. Effectively, one is closer to a brother and an aunt compared to unknown inbreeding individuals. Thus, since most of the time people want to preferentially help their kin (the “kin selection”), our results suggest that they do not avoid them and continue to promote their well-being even if they consider (“their”) inbreeding people as a “nuisance.” Perhaps considering inbreeding people as harmful individuals induced less costs than avoiding them (which is a more active behavior). Further studies could explore the influence of the costs associated with attitudes and behaviors on participants' responses. For example, judging someone is a cheaper response than doing something to someone, which is a more active reaction. Even among actions, different costs could be incurred: punishing someone with a penalty is costlier than avoiding sitting next to someone. As we examine women's responses only, we are limited to making inferences about a sex-dependent effect. Nevertheless, Antfolk et al.'s (2012b) previous works showed that men feel less disgust about inbreeding than women (regarding the degrees of involvement and relatedness). As emotional reaction is the strongest predictor of moral judgment and behaviors (Haidt, 2007), we might think that men would be more tolerant of inbreeding people: feeling less disgust should lead to a less harsh moral or nuisance judgment. However, we tend to expect that men's avoidance would be the same as women's since what is taken into account is the desire to foster our kin. It would be an interesting perspective to explore.

If you are asked “which couple do you judge as the more intolerable: a brother and sister couple or a cousins one?” you will quickly answer “the sib one:” your aversive reaction is conditioned by the relatedness between the two people involved. This may be explained in two ways: you thought about the cultural relationship between the two people (Fraley and Marks, 2010; Lieberman et al., 2011) and/or you considered the risk of inbreeding depression linked to the shared genes between the inbreeding individuals (Bittles and Neel, 1994; Aoki, 2005). In our study, when faced with an inbreeding story, the higher the degree of relatedness (brother/sister > uncle/niece > cousins) was, the more the emotional, and moral aversion was greater. The participants also had a tendency to consider that inbreeding people were more a nuisance when they were closer to each other. However, our contrasts results did not show that the more the inbreeding people were close to each other genetically speaking, the more the participants wanted to avoid them, but there was an influence of the degree of relatedness on avoidance (Table 2). Actually, the participants tended to avoid inbreeding people less when they were definitely the same age (brother and sister, *r* = 0.5; or cousins, *r* = 0.125) and more when there was a potential doubt about their age difference (uncle and niece, *r* = 0.25) even if our stories pointed out they were about the same age. Therefore, the absence of an effect of the degree of relatedness may be connected to a generational bias (ESM 2 in Supplementary Material) which could be more important when actual kin are involved: people do not want to avoid their own generation. The explanation could also be linked to the other manipulated variable (the degree of involvement). Effectively, the higher the involvement is, the more the degree of relatedness between a participant and the people involved in the story is high, leading to consider the degree of involvement as an indirect degree of relatedness. Thus, it could elicit a bias regarding avoidance in the same way as described above: participants would not choose to avoid their kin.

Finally, this study attempted to highlight an important clue of kinship that is supposed to strongly influence inbreeding aversion (Lieberman et al., 2007; Sznycer et al., 2016). Analyses showed that having lived with another child induced less avoidance attitudes and marginally influenced the negative emotions felt. Although, the duration of coresidence with a same-sex sib significantly influenced the disgust felt, negative emotions, and moral judgment, coresidence with an other-sex sib did not show such a pattern of results. According to the parental investment theory (Trivers, 1972), these results might be explained by the fact that having a same-sex sib (i.e., a sister in our study) involved in inbreeding intercourse incurred more costs compared to having an other-sex sib involved. Effectively, one's sister will risk more resources than a brother if the inbreeding intercourse results in a child. Furthermore, egocentric empathy (the fact of reacting to others' behaviors as if they were our own) may be more important between people of the same sex (and perhaps especially with kin members) and potentiate the emotions felt. Our data were too small to allow further analyses, but these findings might also result from a bias linked to the involvement of an actual brother or sister in some stories. Although many previous studies (e.g., Lieberman et al., 2007; Sznycer et al., 2016) found an effect of the duration of coresidence on inbreeding aversion, our study tends to suggest that relationships between siblings are probably much more complicated. It could be interesting to test the influence of other parameters such as the degree of emotional closeness with kin members involved in inbreeding or the activities shared by kin on aversive reactions to inbreeding (De Smet et al., 2014).

Previous studies investigating the fitness costs on aversive reactions to inbreeding people focused on the disgust felt but did not consider the possible effects on attitudes and behaviors. To our knowledge, our study is the first to investigate the aversive reaction to inbreeding people from the disgust felt to social avoidance by taking into account different types of responses to incest. The results of the present study suggest that disgust can be a powerful variable to investigate the influence of fitness costs, but it may fail to account for interesting nuances in aversive reactions (especially regarding the different ways of behaving when faced with inbreeding people and the costs linked to each behavior). This study also showed that if the participants are very disgusted at first, they hardly endorse “punitive” behavior toward inbreeding people, and are even less likely to do so when the inbreeding people are their own kin. More fundamentally, our data demonstrate the utility of considering behaviors and attitudes, not only for our understanding of reactions to inbreeding people, but also for our understanding of reactions to kin members involved in moral or social transgressions.

## Ethics statement

This study was conducted in accordance with the ethical standards of the Institutional and National Guidelines and with the Declaration of Helsinki (2008). Informed consent was obtained from all individual participants included in the study.

## Author contributions

Conceived and designed the experiments: FL and GK. Performed the experiments: FL. Analyzed the data and wrote the paper: FL and GK.

## Funding

This work was supported by grants from the French National Research Agency (GK, grant n°ANR-13-JSH2-0006).

### Conflict of interest statement

The authors declare that the research was conducted in the absence of any commercial or financial relationships that could be construed as a potential conflict of interest.
