# Supplementary material for: Fitness Costs Predict Emotional, Moral, and Attitudinal Inbreeding Aversion
Source: Front Psychol. 2016 Nov 24;7:1860. doi: 10.3389/fpsyg.2016.01860 (PMC5121126; doi:10.3389/fpsyg.2016.01860)
Supplement: Supplementary file 1 [file Presentation1.PDF]

Electronic Supplementary Material (ESM)

Fitness costs predict emotional, moral and attitudinal inbreeding aversion

Florence Lespiau*, Gwenaël Kaminski

*** Correspondence:** Corresponding Author: florence.lespiau@univ-tlse2.fr

ESM 1: Participants

A total of 192 people answered our survey. 33 men were excluded from the analyses as they were too few and are supposed to feel lower aversion; 19 women were excluded because they had experienced an inbreeding relationship and may have been biased. Our 140 female participants ranged in age from 18 to 67 years (mean age (±SD): 24.1 (±9.7)) (Table S1). 78.6% (N=110) were raised by their mother and father. 11.4% (N=16) were only children, 35% (N=49) were firstborns and 53.6% (N=75) were laterborns. 61.4% (N=86) had at least one brother and 55% (N=77) had at least one sister (Table S2). 88.6% (N=124) lived with a brother or a sister. The longest coresidence with an other-sex sibling was 15.2 (±5.0) years and 14.5 (±4.5) years with a same-sex sibling (Table S3). 91.7% (N=131) had at least one uncle and 90.3% (N=129) had at least one aunt (Table S4). 89.6% (N=128) had at least one male cousin and 88.2% (N=126) had at least one female cousin (Table S5). Most participants had a relationship (Table S6), did not have children (Table S7) and were strictly heterosexual 83.6% (N=117). 79.3% (N=111) of them were students.

Table S1 : Age distribution of participants.

Age	N	%
18-20	73	52.1
21-25	42	30.1
26-30	10	7.1
31-35	2	1.4
36-40	2	1.4
41-45	1	0.7
46-50	3	2.1
51-55	3	2.1
56-60	3	2.1
61-67	1	0.7

Table S2 : Siblings distribution among the participants.

Number of brothers	N	%	Number of sisters	N	%
0	54	38.6	0	63	45.0
1	56	40.0	1	56	40.0
2	24	17.1	2	16	11.4
3	1	0.7	3	4	2.9
4	4	2.9	4	0	0
5	1	0.7	5	1	0.7

Table S3 : Longest coresidence (in years) with a sib.

Coresidence with brother	N	%	Coresidence with sister	N	%
0	58	41.4	0	71	49.7
1-5	4	2.8	1-5	4	2.8
6-10	10	7.0	6-10	8	5.6
11-15	18	12.6	11-15	24	16.8
16-20	46	32.2	16-20	33	23.1
21-25	4	2.8	21-25	0	0

Table S4 : Uncle and aunt distribution among the participants.

Number of uncle	N	%	Number of aunt	N	%
0	9	6.4	0	11	7.9
1	25	17.9	1	21	15.0
2	29	20.7	2	27	19.3
3	25	17.9	3	23	16.4
4	52	37.1	4	58	41.4

Table S5 : Cousins distribution among the participants.

Number of male cousin	N	%	Number of female cousin	N	%
0	12	8.6	0	14	10.0
1	23	16.4	1	17	12.1
2	18	12.9	2	16	11.4
3	15	10.7	3	16	11.4
4	72	51.4	4	77	55.0

Table S6 : Relationship status among the participants.

Relationship status	N	%
In relationship with someone	86	61.4
Without current relationship	43	30.7
“Hard to say”	11	7.9

Table S7 : Number of child among the participants.

Number of child	N	%
0	123	87.9
1	7	5.0
2	6	4.3
3	3	2.1
4	1	0.7

ESM 2: How stories were assigned according to participants’ actual kin

The choice regarding which story was presented was based on the participants’ actual kin. For example, if Jessie (one of our participants) is an only child, she was not presented with stories

concerning the intermediate degree of involvement (where one of her sibs is involved). Similarly, under conditions of high (where the participant is personally involved) and intermediate degrees of involvement, the characters of the story were individuals who actually exist for the participant. If one participant did not have any kin, she was presented with a third-party unrelated inbreeding story (see Table S8 for cross-modality effectives). Only 6 out of 52 participants did not have any siblings and they were given the story involving unknown inbreeding people. This allowed us to think that the manipulated effect of relatedness was not an effect of overall sensitivity to inbreeding on the participants.

We used stories featuring a between-generational couple (uncle-niece or aunt-nephew, degree of relatedness r=0.25) while the other stories (degree of relatedness r=0.5 and r=0.125 for intercourse involving brother-sister and cousins respectively) described within-generational couples. Between- and within-generational couples involve different degrees of relatedness in a qualitative way and between-generational inbreeding induces stronger aversion for parent-child intercourse (degree of relatedness r=0.5) (Antfolk et al., 2012a). Effectively, we should have used half-sibling inbreeding intercourse for the degree of relatedness r=0.25. But we expected more people to have an aunt and an uncle rather than a half-sib, and as the participants were presented with stories involving their actual behavior, we wanted to obtain as many equivalent groups as we could and keep the between-generational couple. Effectively, according to INSEE demographic studies, among the French population, there were more than 5 million families with at least 2 children in 2007 and 2012. In 2011, 1.5 million children under 18 lived in 720 000 stepfamilies and only 410 000 children lived with half-siblings (genetically speaking) (Lapinte, 2013).

Table S8 : Effectives for each cross-modality regarding the two main variables (degree of involvement and degree of relatedness).

		Degree of involvement			
		High	Intermediate	Low	Total
Degree of relatedness	r=.5	10	18	15	43
	r=.25	9	19	16	44
	r=.125	13	20	20	53
Total		32	57	51	140

ESM 3: Details regarding dependent variables and accessory dependent variables

Disgust. “How intensely do you feel disgust?” was measured on a visual analogue scale (VAS) from 0 (“you do not feel this emotion at all”) to 100 (“you feel this emotion intensely”). The *disgust* variable was used to confirm previous research that preferentially used disgust as a dependent variable (see ESM4 for more information about *disgust*).

Negative emotions. “How intensely do you feel these emotions?” was measured on a visual analogue scale (VAS) from 0 (“you do not feel this emotion at all”) to 100 (“you feel this emotion intensely”) for each of the following emotions: disgust, compassion/sympathy (reversed item), shame, fear, amusement/laughter (reversed item), confusion/trouble/discomfort, sadness, guilt. The negative emotions variable was used to complement previous research that preferentially used disgust as the only emotion measured.

Moral judgment. "Do you think this behavior is moral?" was measured on a VAS from 0 ("not moral at all") to 100 ("quite moral").

Nuisance. "Do you think this behavior [inbreeding] causes a nuisance to…?" was measured on a VAS from 0 ("not at all") to 100 ("quite a lot") regarding "their close circle (family, friends, etc.)" [inbreeding people's close circle], "society", "themselves" [inbreeding people].

Avoidance attitude. "Now that you have just learned about it [inbreeding intercourse], will you lend them money in the same way as before?" and "Now that you have just learned about it [inbreeding intercourse], could you attend get-togethers with them?" were measured on a VAS from 0 ("no, definitely not") to 100 ("yes, absolutely") for each individual involved in the inbreeding story (except for the descriptions involving the participant herself which only included the other inbreeding individual).

For each multiple VAS variable, the VAS scores for each item were averaged to obtain a single measure.

First emotion felt. After reading the story, the participants were asked to write their first emotion felt. When faced with inbreeding stories, the participants mainly felt disgust (54.2%, N=76) and incomprehension/surprise (12.8%, N=18) while they reported indifference (53.6%, N=75) and amusement/joy (18.8%, N=26) when faced with non-inbreeding stories.

Non-inbreeding vs. inbreeding stories. Each of the four variables used was highly significant between the two stories: the participants showed significantly (*ps*<.001) stronger negative emotions, judged the intercourse as less moral, considered that the two individuals involved were a greater nuisance, and that they were less likely to continue their interactions with them when the story was an inbreeding one compared to a non-inbreeding story. Thus, analyses focused on individual differences regarding the inbreeding story.

Punishment. We initially asked in the inbreeding story what punishment the couple should receive. Nevertheless, 89.3% (N=125) of the participants judged there was no need for punishment, so we did not explore or analyze the factor.

ESM 4: Categories of disgust for ordinal logistic regression and statistical analyses

People often have a split reaction to inbreeding: they are quite tolerant or not at all tolerant. As the *disgust* variable was not normally distributed, we wanted to highlight this cultural separation by dividing the disgust felt into three categories based on the level of disgust felt: low disgust felt (N=36, first quarter), intermediate disgust felt (N=68, second and third quarters) and high disgust felt (N=36, fourth quarter) (Table S9). We then conducted ordinal logistic regression.

Table S9 : Disgust distribution among participants facing an inbreeding intercourse (from 0 to 100).

Disgust	N	%
0-12 (low)	36	25.7
13-96 (intermediate)	68	48.6
97-100 (high)	36	25.7

As we did not have any hypotheses about the interaction between the degree of involvement and the degree of relatedness, we only conduced tests to investigate our assumptions.

References

Lapinte, A. (2013). Un enfant sur dix vit dans une famille recompose. INSEE Première, 1470, 1-4. [Online, 12/16/2015] http://www.insee.fr/fr/themes/document.asp?ref_id=ip1470

INSEE. RP2007 and RP2012. [Online, 12/16/2015] http://www.insee.fr/fr/themes/dossier_complet.asp?codgeo=FE-1
